# Reduction in creatine metabolites in macrophages exposed to small molecule analogues of the anti‐inflammatory parasitic worm product ES‐62

**DOI:** 10.1111/pim.13026

**Published:** 2024-02-19

**Authors:** S. Alanazi, J. Doonan, F. E. Lumb, N. Alenzi, S. Jabbar, L. Al‐Riyami, C. J. Suckling, W. Harnett, D. G. Watson

**Affiliations:** ^1^ King Saud University, College of Applied Medical Sciences, Clinical Laboratory Sciences Department Riyadh Saudi Arabia; ^2^ Strathclyde Institute of Pharmacy and Biomedical Sciences, University of Strathclyde Glasgow UK; ^3^ Research and Laboratories Sector, National Drug and Cosmetic Control Laboratories (NDCCL), Saudi Food and Drug Authority Riyadh Saudi Arabia; ^4^ Department of Biology University of Kirkuk, College of Science Kirkuk Iraq; ^5^ Department of Pure and Applied Chemistry University of Strathclyde Glasgow UK

**Keywords:** CpG, creatine, ES‐62, helminth, metabolomics, nematode, phosphorylcholine

## Abstract

ES‐62, a protein secreted by *Acanthocheilonema viteae*, is anti‐inflammatory by virtue of covalently attached phosphorylcholine (PC) residues and thus a library of drug‐like small molecule analogues (SMAs) based on its PC moieties has been designed for therapeutic purposes. Two members, SMAs 11a and 12b, were previously found to suppress production of pro‐inflammatory cytokines by mouse bone marrow‐derived macrophages (BMMs) exposed to cytosine‐phosphate‐guanosine oligodeoxynucleotides (CpG), agonists for Toll‐like receptor 9. In order to explore the mechanism of action underlying such activities, an untargeted mass spectrometry‐based metabolomics screen was undertaken. Stimulation of BMMs with CpG produced significant metabolic changes relating to glycolysis and the TCA cycle but the SMAs had little impact on this. Also, the SMAs did not promote alterations in metabolites known to be associated with macrophage M1/M2 polarization. Rather, BMMs exposed to SMAs 11a or 12b prior to CpG treatment, or even alone, revealed downregulation of metabolites of creatine, a molecule whose major role is in the transport of high energy phosphate from the mitochondria to the cytosol. These data therefore provide insight into a possible mechanism of action of molecules with significant therapeutic potential that has not previously been described for parasitic worm products.

## 1 INTRODUCTION

ES‐62 is a protein secreted by *Acanthocheilonema viteae*, which is anti‐inflammatory by virtue of covalently attached phosphorylcholine (PC) residues (reviewed in^1^). ES‐62 prevents disease development and/or progression in mouse models of allergy, autoimmunity and cardiovascular disease,^2^, ^3^, ^4^, ^5^, ^6^ suggesting that it has therapeutic potential against a number of the major forms of non‐communicable disease that afflict humans in the 21st century. However, as a large, ‘foreign’, and hence likely immunogenic molecule, ES‐62 is unsuitable for development as a drug. For this reason, we designed a library of synthetic Small Molecule Analogues (SMAs) based around ES‐62's active PC moiety and have identified two molecules—SMA 11a and 12b—that mimic the parent molecule's properties.^7^, ^8^, ^9^ These SMAs are much more suitable for drug development as in addition to being non‐immunogenic, they are cheap and easy to produce, have no toxic liabilities, and are active against human cells in vitro.^10^

In recent years, there has been great interest in exploring the metabolic changes that result from the activation of immune cells, including the macrophage.^11^, ^12^, ^13^ Thus, as an approach to understanding SMA‐mechanism of action we investigated the effect of SMAs 11a and 12b on the metabolome of mouse BMMs both resting and exposed to cytosine‐phosphate‐guanosine oligodeoxynucleotide (CpG). The latter PAMP drives pro‐inflammatory cytokine production via TLR9 and we choose it as this TLR employs the known SMA target, MyD88,^10^ as an adaptor protein and indeed we have previously shown such cytokine production to be inhibited by the SMAs in BMMs.^7^

## 2 MATERIALS AND METHODS

### 2.1 Chemicals and reagents

#### 2.1.1 Treatment of bone marrow‐derived macrophages (BMMs) and preparation of cell extracts

The work was undertaken with the approval of and in accordance with the Home Office UK and the Ethics Review Board of the University of Strathclyde. Bone marrow (BM) was collected from the femur and tibia bones of 6–8‐week old male or female BALB/c mice, bred in Strathclyde University, and killed by cervical dislocation. BM cells were plated and cultured on bacteriological Petri dishes at a density of 2 × 10^6^ cells/mL in ‘complete’ DMEM (contains 2 mM glutamine, 50 U/mL penicillin, 50 mg/mL streptomycin and 10% foetal calf serum) with 20% L929 cell supernatant (contains M‐CSF) and maintained at 37°C in a humidified atmosphere of 5% (v/v) CO_2_ as described previously.^7^ Fresh medium was added on day four. On day seven, the cells were harvested by scraping them into 5 mL complete DMEM at 4°C and were then recovered for further plating by centrifugation at 400*g* for 5 min. The viability and number of cells was checked using trypan blue stain followed by identification by flow cytometry using a FACS Canto (BD Pharmingen) in concert with antibodies against CD11b (CD11b‐APC; BD Pharmingen) and F4/80 (F4/80‐FITC; eBioscience) as described previously.^7^ Cultures of >99% purity for CD11b+F4/80+ cells were considered BM‐derived macrophages (BMM) and were used for metabolomics assays.

BMMs were employed at 2 × 10^6^ cells/2 mL of ‘complete’ RPMI medium,^7^ in 6‐well plates. SMAs 11a, 12b and 19o, prepared as described previously^7^ were added at 5 μg/mL and BMMs incubated for 18 h at 37°C in a humidified atmosphere of 5% (v/v) CO_2_ (*n* = 5). In some experiments, incubation was continued for a further 24 h (*n* = 6) in the presence of, CpG, (0.1 μM; CpG‐ODN1826; InvivoGen), LPS (100 ng/mL; *Salmonella minnesota*; Sigma–Aldrich) IFN‐γ (100 U/mL; Sigma–Aldrich), IL‐4 (100 U/mL; eBioscience) or combinations of these reagents.

Cell extracts were prepared by washing with PBS before harvesting the cells in a chilled extraction solution (MeOH/MeCN/H_2_O, 50:30:20 v/v) with a concentration of 1 mL of extraction mix per 2 × 10^6^ cells. Cell lysates were collected and shaken for 20 min at 4°C before being centrifuged at 0°C at 13,000 rpm for 15 min. The supernatants were then collected and transferred into auto sampler vials for loading into the LC–MS autosampler or storage at −80°C until analysis.

#### 2.1.2 Liquid chromatography/mass spectroscopy (LC/MS)

The chromatographic conditions were set as follows: A ZICpHILIC column (150 × 4.6 mm × 5 μm, Hichrom, Reading, UK) was eluted with a linear gradient over 30 min between 20 mM ammonium carbonate (pH 9.2)/MeCN (20:80) at 0 min and 20 mM ammonium carbonate (pH 9.2)/MeCN (80:20) at 30 min with a flow rate of 0.3 mL/min, followed by washing with 20 mM ammonium carbonate MeCN (95:5) for 5 min and then re‐equilibration with the starting conditions for 10 min. LC/MS was carried out by using a Dionex 3000 HPLC pump coupled to an Exactive (Orbitrap) mass spectrometer from Thermo Fisher Scientific. The spray voltage was 4.5 kV for positive mode and 4.0 kV for negative mode. The temperature of the ion transfer capillary was 275°C and sheath and auxiliary gas were 50 and 17 arbitrary units, respectively. The full scan range was 75–1200 m/z for both positive and negative modes. The data were recorded using the Xcalibur 2.1.0 software package (Thermo Fisher Scientific). The signals of 83.0604 m/z (2 × ACN + H) and 91.0037 m/z (2 × formate‐H) were selected as lock masses for the positive and negative modes, respectively, during each analytical run.

#### 2.1.3 Metabolomic data analysis

Raw data from untargeted metabolomic studies, were putatively identified and processed using Mzmine.^14^ Prior to further analysis, data were filtered so that metabolite peaks with low intensities (<1000 peak height) were excluded. Accurate masses were searched against an in‐house database and in addition, retention times were matched for 200 metabolites against standard mixtures run at the same time as the samples.^15^ Otherwise, metabolite matches were based on accurate masses <3 ppm and thus putatively identified to MSI level 2.^16^ The data from Mzmine was transferred to Xcel and ratios and P values were determined by using Microsoft Excel. Metabolites which did not show any significant differences were excluded in order to simplify the data for interpretation.

## 3 RESULTS AND DISCUSSION

SMAs were employed at 5 μg/mL, a concentration we had previously shown to inhibit pro‐inflammatory cytokine production by CpG‐activated macrophages, but to have no effect on cell viability.^7^ SMAs were first tested in the absence of GpG stimulation and Table 1 shows that exposure of known immunomodulatory SMAs 11a and 12b to BMMs only induced a small number of metabolic alterations, ~50 and ~25, respectively, with all changes induced by 12b also induced by 11a. Only three changes were observed with SMA 19o, a structurally related but relatively inactive SMA.^7^ The changes associated with the two active SMAs included alterations in the levels of a few metabolites scattered across a number of categories of molecule and biosynthetic pathway including taurine metabolism, choline metabolism, oxidative stress, carnitines and carnitine biosynthesis, and in particular phospholipids. However, potentially the most interesting change related to creatine metabolism, where each of guanidinoactetate, creatine and phosphocreatine where significantly reduced by the two active SMAs, 11a and 12b, but not by the relatively inactive SMA 19o.

**TABLE 1:** The list of detected metabolites that have changed following treatment with one or more of SMAs 11a, 12b or 19o in comparison to untreated BMMs (statistical analysis by Students *t* test, significant differences highlighted in bold font).

DM	m/z	RT	Name	11a P	11a F	12b P	12b F	19o P	19o F
Oxidative stress									
+	613.200	17.8	Glutathione disulphide	**<0.001**	**1.282**	**<0.001**	**4.143**	0.289	1.152
+	336.100	14.9	S‐Formylglutathione	**0.007**	**0.808**	**<0.001**	**0.635**	**0.016**	**1.199**
+	166.100	13.8	L‐Methionine S‐oxide	**0.006**	**0.806**	0.115	0.826	0.699	1.044
+	241.000	16.7	L‐Cystine	**0.01**	**0.638**	**<0.001**	**0.329**	0.35	0.873
Taurine metabolism									
+	110.000	15.3	Hypotaurine	**<0.001**	**0.538**	**<0.001**	**0.451**	0.781	0.989
−	124.000	15.2	Taurine	**<0.001**	**0.743**	**<0.001**	**0.679**	0.471	0.983
+	126.000	15.2	Taurine	**<0.001**	**0.687**	**<0.001**	**0.594**	0.137	1.036
Choline metabolism									
+	184.100	15.4	Choline phosphate	**0.005**	**1.143**	0.119	1.104	0.649	1.034
+	258.100	14.9	sn‐glycero‐3‐Phosphocholine	**<0.001**	**0.644**	**<0.001**	**0.466**	0.168	0.943
Carnitines and carnitine biosynthesis									
+	147.100	25.3	L‐Lysine	**0.04**	**0.855**	0.484	0.914	0.536	1.075
+	146.100	13.7	4‐Trimethylammoniobutanoate	**<0.001**	**0.683**	**<0.001**	**0.358**	0.478	0.977
+	162.100	13.7	L‐Carnitine	**<0.001**	**0.715**	**<0.001**	**0.531**	0.297	1.066
+	204.100	11.2	O‐Acetylcarnitine	**<0.001**	**0.685**	**<0.001**	**0.414**	**0.023**	**0.915**
+	232.200	9.0	O‐Butanoylcarnitine	**<0.001**	**0.84**	**0.014**	**0.821**	0.451	0.944
+	372.300	4.9	Tetradecanoylcarnitine	**<0.001**	**0.781**	**<0.001**	**0.675**	0.56	1.018
Purine and pyrimidine metabolism									
+	228.100	10.8	Deoxycytidine	**0.01**	**0.793**	**<0.001**	**0.455**	0.611	0.966
+	263.100	8.2	Thiamine aldehyde	**0.024**	**0.785**	0.304	0.862	0.46	0.908
Aminosugar metabolism									
+	310.100	13.6	N‐Acetylneuraminate	**0.016**	**0.932**	**0.002**	**0.868**	0.155	1.063
−	308.100	13.6	N‐Acetylneuraminate	**0.02**	**0.917**	**0.002**	**0.848**	0.058	1.133
Glycolysis and TCA cycle and related metabolites									
−	179.100	17.4	D‐Glucose	**0.002**	**0.749**	0.113	0.802	0.644	1.037
−	171.000	14.9	sn‐Glycerol 3‐phosphate	**<0.001**	**0.621**	**<0.001**	**0.418**	**0.004**	**0.873**
+	170.100	8.2	Pyridoxine	**0.013**	**0.812**	0.498	0.916	0.823	0.977
+	664.100	14.6	NAD+	**0.004**	**1.073**	**<0.001**	**1.255**	0.549	1.026
Creatine metabolism									
−	210.000	15.5	Phosphocreatine	**<0.001**	**0.579**	**<0.001**	**0.477**	0.957	1.003
+	118.100	16.1	Guanidinoacetate	**<0.001**	**0.801**	**<0.001**	**0.445**	0.507	0.973
+	132.077	15.5	Creatine	**0.014**	**0.482**	**<0.001**	**0.534**	0.148	1.153
Miscellaneous									
+	205.100	12.1	L‐Tryptophan	**0.021**	**0.852**	0.434	0.912	0.725	1.037
+	150.100	11.9	L‐Methionine	**0.022**	**0.845**	0.477	0.921	0.868	1.017
+	175.100	13.9	N‐Acetylornithine	**0.017**	**0.83**	0.499	0.916	0.884	1.018
−	145.100	25.3	L‐Lysine	**0.026**	**0.829**	0.406	0.907	0.687	1.041
+	176.100	4.4	Indole‐3‐acetate	**0.035**	**0.814**	0.444	0.906	0.844	0.978
−	218.100	8.8	Pantothenate	**0.011**	**0.805**	0.423	0.897	0.902	0.986
−	181.100	14.2	D‐Sorbitol	**0.002**	**0.756**	0.285	0.869	0.042	1.284
−	164.100	10.6	L‐Phenylalanine	**0.015**	**0.751**	0.056	0.767	0.1	0.822
Phospholipids									
−	245.000	13.0	Glycerophosphoglycerol	**<0.001**	**0.753**	**<0.001**	**0.759**	0.295	1.076
+	247.100	12.9	Glycerophosphoglycerol	**<0.001**	**0.781**	**<0.001**	**0.755**	0.901	1.005
+	216.100	16.0	sn‐glycero‐3‐Phosphoethanolamine	**<0.001**	**0.596**	**<0.001**	**0.501**	0.289	0.959
−	214.000	16.0	sn‐glycero‐3‐Phosphoethanolamine	**<0.001**	**0.583**	**<0.001**	**0.489**	0.514	0.969
+	752.600	4.0	PE38:5	**<0.001**	**1.215**	0.08	1.083	0.167	0.928
+	766.600	4.2	PC36:4 ether	**<0.001**	**1.178**	0.073	0.941	0.706	1.015
−	437.300	3.9	[GP (18:0)] 1‐octadecanoyl‐2‐sn‐glycero‐3‐phosphate	**0.004**	**1.168**	0.237	1.071	0.659	1.04
−	838.600	3.8	1‐22:1‐2‐18:3‐phosphatidylserine	**0.004**	**1.153**	0.36	1.05	0.301	0.93
+	788.600	4.2	PC36:1	**0.004**	**1.133**	**0.041**	**1.118**	0.137	0.926
−	786.500	3.9	PS36:2	**0.004**	**1.121**	0.817	1.01	0.226	0.939
+	718.600	4.2	PC32:0 ether	**0.001**	**1.112**	**<0.001**	**1.224**	0.192	0.96
+	840.600	3.8	PS40:4	**0.012**	**1.107**	0.172	0.944	0.29	0.935
+	703.600	4.4	[SP (16:0)] N‐(hexadecanoyl)‐sphing‐4‐enine‐1‐phosphocholine	**0.036**	**1.104**	0.763	1.014	0.316	0.938
+	792.600	4.2	PC38:5 ether	**0.025**	**1.084**	0.845	0.993	0.964	1.002
−	746.500	4.1	PE38:7	**0.007**	**1.069**	**0.047**	**0.94**	0.957	0.999
+	482.300	4.7	Lyso PE 18:0	**0.048**	**0.936**	**0.015**	**0.875**	0.409	1.056
+	149.100	15.5	[FA methyl,hydroxy(5:0)] 3R‐methyl‐3,5‐dihydroxy‐pentanoic acid	**0.023**	**0.862**	0.324	0.909	0.711	0.972
−	738.500	4.1	PE 36:4	**<0.001**	**0.854**	**<0.001**	**0.893**	0.449	1.02
−	498.300	4.4	[ST hydrox] N‐(3alpha,7alpha‐dihydroxy‐5beta‐cholan‐24‐oyl)‐taurine	**0.01**	**0.831**	0.463	0.919	0.484	0.941

*Note*: DM refers to detection mood, m/z to mass to charge ratio, F to fold change, RT to raw retention time and P to *p*‐values. Data are from a single experiment (*n* = 5 technical replicates) and are representative of three experiments in total.

Treating macrophages with CpG for 24 h alters the levels of a multitude of metabolites in a number of pathways (Table S1), the observed changes being broadly in line with those observed previously for macrophages treated with lipopolysaccharide.^17^ In many cases treating the BMMs with either of the two active SMAs prior to CpG treatment led to changes in the levels of metabolite although this was often also seen with SMA 19o (Table S2). Based on the aforementioned LPS‐macrophage studies, glycolysis and the TCA cycle have been presented as possible targets for intervention in the control of the inflammatory response. However, only a few metabolites in these pathways were affected by the SMAs (Table S2). Nevertheless, as with resting cells, pre‐treatment of the CpG‐activated macrophages with the two active SMAs led to significant changes in levels of each of guanidinoacetate, creatine and phosphocreatine. All of these are increased in CpG‐treated cells (Table S2), but are lowered in the presence of the two active SMAs (Table S2 and highlighted in Table 2). As with other metabolites, there was also a reduction in creatine metabolites with SMA 19o but this was to a much lesser extent.

**TABLE 2:** Effect of SMA pre‐treatment on creatine metabolites in CpG‐exposed cells (the changes for the CpG exposure alone are expressed relative to the medium control).

						CpG	CpG	CpG	CpG	CpG	CpG
DM	m/z	RT	Name	CpG P	CpG F	11a P	11a F	12b P	12b F	19o P	19o F
+	118.061	16.0	Guanidinoacetate	<0.001	2.923	<0.001	0.238	<0.001	0.540	<0.001	2.026
+	132.077	15.0	Creatine	<0.001	2.499	<0.001	0.613	<0.001	0.625	<0.001	1.734
+	212.043	15.4	Phosphocreatine	<0.001	3.667	<0.001	0.573	<0.001	0.622	0.196	1.080

*Note*: DM refers to detection mode, m/z to mass to charge ratio, RT retention time (min), F to fold change and P to *p*‐values. Data are from a single experiment (*n* = 5 technical replicates) and are representative of two experiments in total.

**FIGURE 1:**
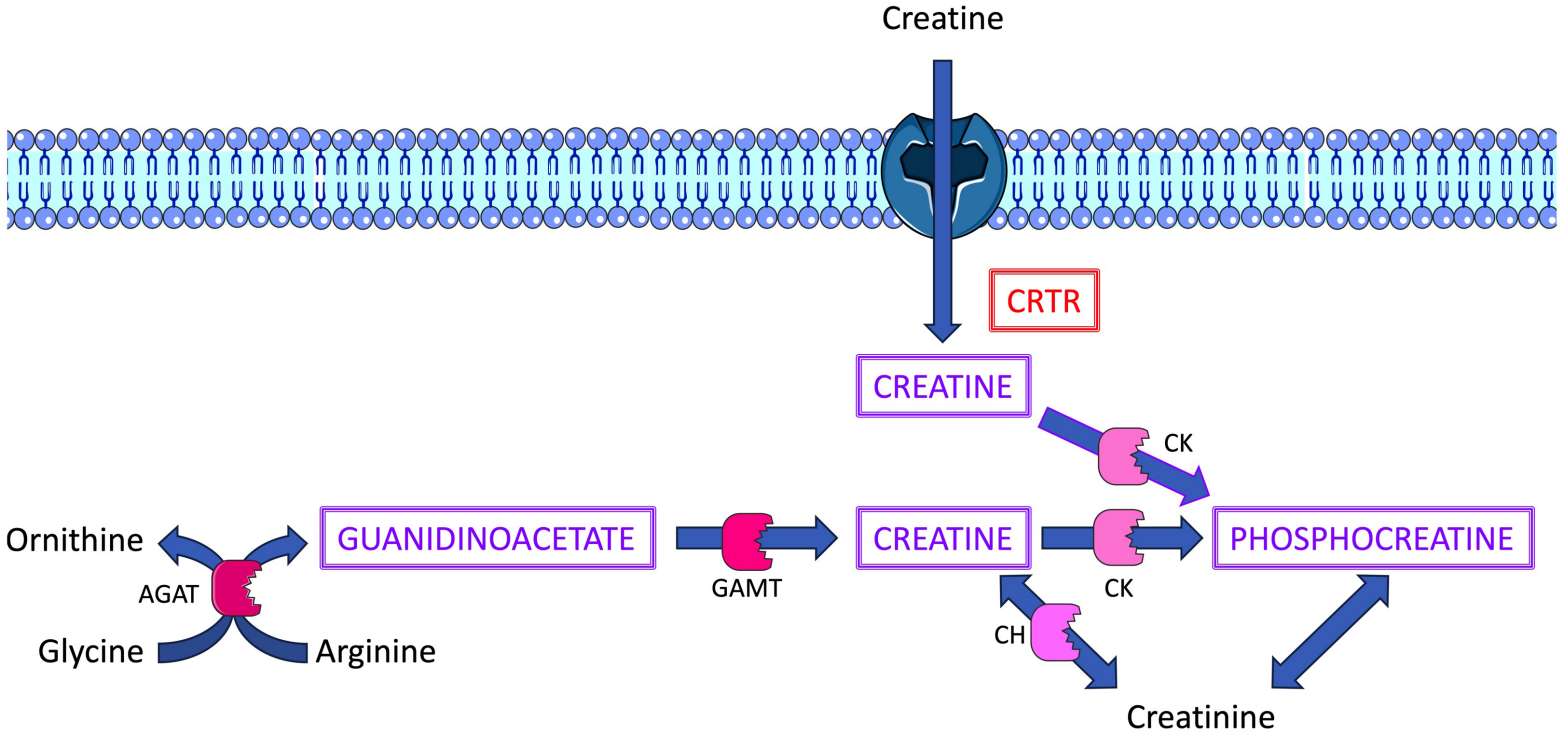
Graphical representation of effect of ES‐62 SMAs 11a and 12b on creatine uptake and metabolism in mouse BMMs. Creatine is mainly produced by the liver and taken up by other cells via a specific transporter (CRTR, red font and boxed). It is considered likely that the SMAs reduce creatine uptake via the transporter by a yet unknown mechanism resulting in decreased creatine and phosphocreatine (both upper case, purple font, boxed) within the cell. The reduction in guanidinoacetate (upper case, purple font, boxed) may reflect an attempt by the macrophage to compensate for reduced creatine uptake by increased synthesis. AGAT, arginine:glycine aminidinotransferase; CH, creatine hydrolase; CK, creation kinase; GAMT, guanidineacetate methyltransferase. The figure was partly generated using Servier Medical Art, licensed under a Creative Commons Attribution 3.0 unported licence.

Creatine is converted to phosphocreatine within cells and the latter plays a role in regeneration of ATP via the transport of high energy phosphate from the mitochondria to the cytosol. As far as we aware, modifying creatine metabolism as opposed to oxidative phosphorylation or glycolysis has not previously been described with respect to pathogen products or synthetic molecules based on them. Creatine is generally synthesized by the liver and so it has to be taken up from the bloodstream by the tissues that utilize it. The uptake of creatine by macrophages is rapid and it has been proposed that macrophages have a high requirement for creatine in order to form phosphocreatine, which is required, for example, for phagocytosis.^18^ It is thus likely that uptake of creatine from the culture medium is being affected by the SMAs rather than its biosynthesis. Guanidinoacetate can be employed in the biosynthesis of creatine but macrophage levels of genes involved in creatine biosynthesis are low.^18^ However, guanidinoacetate levels are also lower in the SMA‐treated macrophages and so this could indeed perhaps be due to increased biosynthesis of creatine from guanidinoacetate to compensate for reduced uptake of creatine. A summary of the effect of SMAs 11a and 12b on creatine metabolism and also the likely effect on creatine uptake is shown in Figure 1.

The mechanism by which the SMAs affect creatine uptake is yet to be determined. What also remains to be established is whether the effect of the two active SMAs, 11a and 12b, on creatine levels is related to their inhibitory effects on pro‐inflammatory cytokine production. Certainly, there is much less effect on creatine metabolite levels with the SMA 19o, previously shown to have very little effect on BMM cytokine production.^7^ It has recently been shown that ablation of the creatine transporter Slc6a8, which is highly expressed in mouse BMMs, leads to an almost complete block in creatine uptake and resultant depletion of intracellular pools.^19^ Examination of these cells for changes in immune function led to the conclusion that creatine blocks M1 polarization (decreased *iNOS*) but promotes M2 polarization (e.g. increased *arg1*). Our analysis of the metabolome of BMMs exposed to either M1 (IFN‐γ ± LPS) or M2 (IL‐4)‐polarizing conditions reveals a totally dissimilar metabolome to that associated with the SMAs but rather changes that might be expected, for example, increases in metabolites linked to glycolysis and oxidative phosphorylation with the former, and upregulated L‐ornithine with the latter (Table S3). Thus, the SMAs do not appear to be affecting macrophage polarization by the changes in creatine metabolites that they induce. Also, in our study, overall ATP content of the cells treated with SMAs are similar to those treated with CpG alone (as shown by ATP not being listed in Table S2) and so the effect of the SMAs is not on ATP levels per se but may rather be on the rate of supply of ATP to where it is needed. Could one place where it is needed be in production of pro‐inflammatory cytokines? Certainly, a study in which macrophages were obtained from tumours of mice fed increased creatine, revealed elevated levels of pro‐inflammatory cytokines TNF, IL‐6, IL‐12 and IL‐1β.^20^ This raises the possibility that the inhibitory effects of the SMAs on pro‐inflammatory cytokines could indeed reflect a loss of creatine uptake. However, confirmation of this requires further exploration.

## AUTHOR CONTRIBUTIONS

The study was planned and conceived by DGW, WH and SA. The work was undertaken by SA supported by JD, FEL, NA, SJ and LAR. The SMAs and advice on their use were provided by CJS. WH and DGW drafted the manuscript and all authors were involved it critically reviewing it and approving the final version for publication.

## CONFLICT OF INTEREST STATEMENT

The authors declare no conflicts of interest.

## Supporting information


**Data S1.** Supporting Information

## Data Availability

Excel sheets showing the raw metabolomics data are available on the University of Strathclyde's ‘Pure’ site.
