# Supplementary material for: Reduction in creatine metabolites in macrophages exposed to small molecule analogues of the anti‐inflammatory parasitic worm product ES‐62
Source: Parasite Immunol. 2024 Feb 19;46(2):e13026. doi: 10.1111/pim.13026 (PMC11475200; doi:10.1111/pim.13026)
Supplement: Supplementary file 1 — Data S1. Supporting Information [file PIM-46-e13026-s001.docx]

**Table ‎S1**: The list of metabolites that have changed following CpG treatment of BMMs. DM refers to detection mode, m/z to mass to charge ratio, F to fold change, RT retention time (min) and P to P-value (n=6). * Indicates retention time corresponding to analytical standard. (PC= phosphatidylcholine, PE= phosphatidylethanolamine, PS = phosphatidylserine, PI= phsophatidylinositol, PG = phosphatidylglycerol, SM = sphingomyelin, SP = sphingosine). Data are from a single experiment (n=5 technical replicates) and are representative of two experiments in total.

| DM | m/z | RT | Name | CpG P | CpG F |
| --- | --- | --- | --- | --- | --- |
| **Glycolysis and TCA cycle** | | | | | |
| - | 89.024 | 9.4 | *(R)-Lactate | <0.001 | 2.498 |
| - | 115.004 | 16.2 | *Fumarate | <0.001 | 2.742 |
| - | 117.019 | 15.3 | *Succinate | <0.001 | 2.038 |
| - | 129.019 | 15.2 | *itaconate | <0.001 | 4.720 |
| - | 133.014 | 16.2 | *(S)-Malate | <0.001 | 2.736 |
| + | 168.066 | 8.1 | Pyridoxal | 0.012 | 1.226 |
| - | 145.014 | 15.7 | *2-Oxoglutarate | <0.001 | 4.484 |
| - | 147.030 | 18.4 | 2-Hydroxyglutarate | 0.006 | 1.841 |
| - | 168.991 | 15.5 | *DL-Glyceraldehyde 3-phosphate | 0.000 | 2.755 |
| - | 168.991 | 16.4 | *Dihydroxy acetone phosphate | 0.683 | 1.094 |
| - | 171.006 | 14.8 | *sn-Glycerol 3-phosphate | <0.001 | 1.570 |
| - | 173.009 | 18.5 | *cis-Aconitate | <0.001 | 2.306 |
| - | 179.056 | 13.7 | *D-Glucose | <0.001 | 2.068 |
| - | 184.986 | 17.3 | *3-Phospho-D-glycerate | <0.001 | 30.158 |
| - | 191.020 | 18.4 | *Citrate | <0.001 | 1.776 |
| + | 170.081 | 8.2 | *Pyridoxine | 0.012 | 1.282 |
| - | 259.022 | 17.1 | *Glucose 6-phosphate | <0.001 | 2.330 |
| - | 338.989 | 16.6 | *D-Fructose 1,6-bisphosphate | 0.000 | 1.526 |

| DM | m/z | RT | Name | CpG P | CpG F |
| --- | --- | --- | --- | --- | --- |
| + | 664.117 | 14.4 | *NAD+ | <0.001 | 3.73 |
| + | 666.132 | 13.6 | *NADH | <0.001 | 3.71 |
| + | 810.133 | 12.6 | *Acetyl CoA | <0.001 | 7.849 |
| **Oxidative stress** | | | | | |
| + | 76.039 | 16.0 | *Glycine | <0.001 | 1.583 |
| - | 120.012 | 16.6 | *L-Cysteine | 0.007 | 0.744 |
| + | 148.06 | 14.9 | *L-Glutamate | <0.001 | 2.248 |
| - | 167.021 | 12.7 | *Urate | 0.007 | 1.277 |
| + | 168.052 | 15.1 | 8-Hydroxyguanine | <0.001 | 5.199 |
| - | 184.002 | 3.9 | O-Phospho-L-serine | 0.008 | 0.089 |
| + | 241.031 | 16.6 | *L-Cystine | 0.008 | 0.75 |
| + | 251.07 | 14.4 | ɣ-Glutamyl-L-cysteine | 0.007 | 9.347 |
| + | 308.091 | 14.6 | *Glutathione | <0.001 | 2.17 |
| - | 386.033 | 19.3 | glutathione-sulfite | 0.001 | 3.868 |
| + | 427.095 | 16.9 | S-glutathionyl-L-cysteine | <0.001 | 3.242 |
| + | 613.16 | 17.6 | *Glutathione disulfide | <0.001 | 4.79 |
| + | 744.083 | 17.1 | *NADP+ | <0.001 | 9.896 |
| + | 746.099 | 17.5 | *NADPH | <0.001 | 3.402 |
| **Taurine metabolism** | | | | | |
| + | 110.027 | 15.3 | Hypotaurine | <0.001 | 11.296 |
| + | 126.022 | 15.1 | *Taurine | <0.001 | 3.923 |
| + | 168.044 | 15.9 | Taurocyamine | <0.001 | 6.188 |
| - | 167.997 | 15.1 | L-Cysteate | <0.001 | 7.006 |
| + | 255.065 | 15.7 | 5-L-Glutamyl-taurine | <0.001 | 8.684 |
| DM | m/z | RT | Name | CpG P | CpG F |
| **Choline metabolism** | | | | | |
| + | 104.107 | 20.8 | *Choline | <0.001 | 1.567 |
| + | 184.073 | 15.3 | *Choline phosphate | <0.001 | 2.338 |
| + | 258.110 | 14.8 | *glycero-3-Phosphocholine | <0.001 | 1.602 |
| **ATP and high energy phosphates** | | | | | |
| + | 212.043 | 15.4 | *Phosphocreatine | <0.001 | 3.667 |
| + | 324.059 | 15.5 | *CMP | 0.012 | 2.712 |
| - | 323.029 | 15.4 | *UMP | <0.001 | 3.150 |
| + | 348.070 | 14.0 | *AMP | 0.001 | 3.295 |
| - | 347.040 | 15.7 | *IMP | <0.001 | 3.680 |
| - | 362.051 | 17.0 | *GMP | 0.000 | 1.537 |
| - | 402.995 | 16.8 | *UDP | <0.001 | 17.538 |
| + | 428.037 | 15.5 | *ADP | <0.001 | 3.235 |
| - | 429.058 | 15.6 | CMP-2-aminoethylphosphonate | <0.001 | 6.575 |
| + | 447.068 | 16.6 | CDP-ethanolamine | <0.001 | 6.165 |
| - | 481.977 | 18.9 | *CTP | <0.001 | 40.084 |
| - | 482.961 | 18.3 | *UTP | <0.001 | 20.169 |
| + | 508.003 | 17.0 | *ATP | <0.001 | 4.322 |
| - | 521.983 | 19.9 | *GTP | 0.002 | 29.142 |
| - | 565.047 | 16.6 | *UDP-glucose | <0.001 | 4.917 |
| + | 574.095 | 12.9 | GDP-3,6-dideoxy-D-galactose | 0.002 | 4.006 |
| - | 579.027 | 19.3 | UDP-glucuronate | <0.001 | 7.065 |
| - | 604.070 | 18.5 | GDP-mannose | <0.001 | 3.358 |
| + | 588.0746 | 17.8 | GDP-L-fucose | 0.101 | 2.059 |
| + | 608.089 | 15.3 | *UDP-N-acetylglucosamine | <0.001 | 8.677 |
| + | 615.155 | 15.5 | CMP-N-acetylneuraminate | <0.001 | 3.125 |

| DM | m/z | RT | Name | CpG P | CpG F |  |
| --- | --- | --- | --- | --- | --- | --- |
| **Carnitines and carnitine biosynthesis** | | | |  |  |  |
| + | 146.118 | 13.6 | Trimethylammoniobutanoate | <0.001 | 1.568 |  |
| + | 162.113 | 13.6 | *L-Carnitine | <0.001 | 1.351 |  |
| + | 189.160 | 22.8 | *N6,N6,N6-Trimethyl-L-lysine | 0.003 | 2.110 |  |
| + | 204.123 | 11.3 | *O-Acetylcarnitine | <0.001 | 2.784 |  |
| + | 218.139 | 10 | O-Propanoylcarnitine | <0.001 | 1.811 |  |
| + | 232.154 | 9 | O-Butanoylcarnitine | <0.001 | 1.703 |  |
| + | 248.149 | 11.6 | Hydroxybutyrylcarnitine | <0.001 | 4.932 |  |
| + | 288.217 | 7.6 | L-Octanoylcarnitine | 0.037 | 1.580 |  |
| + | 372.311 | 4.9 | Tetradecanoylcarnitine | <0.001 | 3.079 |  |
| + | 374.254 | 4.2 | Dodecanedioylcarnitine | 0.011 | 0.278 |  |
| + |  | 4.8 | Hexadecenoylcarnitine | <0.001 | 3.489 |  |
| + | 400.342 | 4.7 | Palmitoylcarnitine | <0.001 | 2.945 |  |
| + | 414.358 | 4.1 | Heptadecanoylcarnitine | 0.023 | 0.507 |  |
| + | 424.342 | 4.7 | Linoelaidylcarnitine | <0.001 | 6.716 |  |
| + | 426.358 | 4.7 | Elaidiccarnitine | <0.001 | 3.489 |  |
| + | 428.374 | 4.6 | Stearoylcarnitine | <0.001 | 1.668 |  |
| **Purine and pyrimidine metabolism** | | | | | |  |
| - | 111.020 | 8.6 | *Uracil | <0.001 | 2.150 |  |
| + | 136.062 | 9.8 | *Adenine | 0.034 | 2.455 |  |
| + | 137.046 | 10.4 | *Hypoxanthine | <0.001 | 0.17 |  |
| DM | m/z | RT | Name | CpG P | CpG F |  |
| - | 151.026 | 11.4 | *Xanthine | <0.001 | 0.611 |  |
| + | 166.072 | 13.1 | 3-Methylguanine | <0.001 | 178.727 |  |
| - | 229.012 | 16.0 | *D-Ribose 5-phosphate | 0.001 | 113.44 |  |
| + | 242.114 | 9.7 | 5-Methyl-2'-deoxycytidine | 0.033 | 1.241 |  |
| - | 243.062 | 10 | *Uridine | <0.001 | 0.691 |  |
| - | 243.062 | 12.2 | pseudouridine | 0.020 | 1.258 |  |
| - | 289.033 | 16.6 | Sedoheptulose 7-phosphate | <0.001 | 17.617 |  |
|  | 287.064 | 16.7 | Phosphoribosylglycinamide | <0.001 | 1377.671 |  |
| + | 446.179 | 3.9 | Tetrahydrofolate | 0.001 | >1000 |  |
| **Aminosugar metabolism** | | | | | |  |
| + | 180.087 | 15.2 | *D-Glucosamine | 0.006 | 23.432 |  |
| + | 260.053 | 15.6 | D-Glucosamine 6-phosphate | <0.001 | 2.870 |  |
| + | 310.113 | 13.5 | N-Acetylneuraminate | <0.001 | 1.983 |  |
| + | 326.108 | 14.6 | N-Glycoloyl-neuraminate | <0.001 | 2.765 |  |
| **Creatine metabolism** | | | | | |  |
| + | 76.039 | 16.0 | *Glycine | <0.001 | 1.583 |  |
| + | 114.066 | 9.9 | *Creatinine | 0.005 | 1.242 |  |
| + | 118.061 | 16.0 | Guanidinoacetate | <0.001 | 2.923 |  |
| + | 132.077 | 15.0 | *Creatine | <0.001 | 2.499 |  |
| DM | m/z | RT | Name | CpG P | CpG FC |  |
| + | 133.097 | 23.8 | *L-Ornithine | 0.007 | 1.226 |  |
| + | 150.058 | 11.8 | L-Methionine | 0.006 | 1.299 |  |
| + | 175.119 | 26.9 | L-Arginine | 0.020 | 1.272 |  |
| **Arginine metabolism** | | | | | |  |
| DM | m/z | RT | Name | CpG P | CpG F |  |
| + | 146.092 | 15.5 | 4-Guanidinobutanoate | 0.012 | 1.164 |  |
| + | 174.087 | 14.9 | 5-Guanidino-2-oxopentanoate | 0.049 | 0.273 |  |
| + | 174.087 | 15.6 | 5-Guanidino-2-oxopentanoate | 0.050 | 1.525 |  |
| + | 175.119 | 26.9 | *L-Arginine | 0.020 | 1.272 |  |
| + | 203.150 | 22.3 | Dimethyl-L-arginine | 0.005 | 1.729 |  |
| + | 291.130 | 17.1 | Arginino succinate | <0.001 | 3.467 |  |
| + | 247.140 | 14.5 | Carboxyethyl-L-arginine | 0.001 | 1.337 |  |
| **Histidine metabolism** | | | | | |  |
| + | 139.050 | 10.8 | *Urocanate | 0.011 | 0.576 |  |
| + | 141.066 | 10.5 | Methylimidazoleacetic acid | <0.001 | 2.491 |  |
| + | 141.066 | 9.5 | Methylimidazoleacetic acid | 0.001 | 1.405 |  |
| + | 156.077 | 14.9 | *L-Histidine | <0.001 | 1.417 |  |
| + | 227.114 | 16.0 | *Carnosine | 0.030 | 1.466 |  |
| **Miscellaneous** | | | | | |  |
| + | 90.055 | 15.6 | *L-Alanine | <0.001 | 8.544 |  |
| + | 104.071 | 5.4 | Aminobutanoate isomer | 0.024 | 0.329 |  |
| + | 118.086 | 11.5 | *L-Valine | <0.001 | 1.358 |  |
| + | 123.055 | 7.6 | *Nicotinamide | <0.001 | 2.03 |  |
| + | 128.071 | 13.5 | 2,3,4,5-Tetrahydropyridine-2-carboxylate | 0.038 | 2.098 |  |
| + | 130.05 | 15.4 | L-1-Pyrroline-3-hydroxy-5-carboxylate | 0.001 | 1.286 |  |
| + | 132.065 | 14.8 | L-Glutamate 5-semialdehyde | 0.024 | 1.288 |  |
| + | 134.045 | 15.2 | *L-Aspartate | <0.001 | 1.297 |  |
| + | 134.06 | 8.2 | Indoxyl | 0.020 | 1.249 |  |
| + | 142.026 | 16.2 | Ethanolamine phosphate | <0.001 | 1.798 |  |
| + | 147.076 | 15.4 | *L-Glutamine | 0.003 | 1.263 |  |
| + | 147.113 | 25.4 | *L-Lysine | 0.029 | 1.225 |  |
| + | 160.133 | 13.6 | DL-2-Aminooctanoicacid | <0.001 | 1.902 |  |
| + | 161.129 | 24.3 | *N6-Methyl-L-lysine | <0.001 | 2.923 |  |
| + | 162.076 | 15.1 | Aminoadipate | 0.043 | 2.681 |  |
| + | 162.076 | 11.3 | Aminoadipate | 0.006 | 6.898 |  |
| + | 166.086 | 10.5 | *Phenylalanine | 0.010 | 1.270 |  |
| + | 174.113 | 5.0 | N-Acetyl-L-leucine | 0.011 | 0.170 |  |
| + | 175.108 | 13.8 | N-Acetylornithine | 0.007 | 1.355 |  |
| + | 177.112 | 5.0 | Dihydroxy-octanoic acid | 0.026 | 0.277 |  |
| + | 178.086 | 7.5 | 5-Hydroxytryptophol | 0.026 | 1.288 |  |
| + | 180.066 | 7.5 | Hippurate | 0.037 | 1.351 |  |
| + | 184.061 | 4.8 | 4-Pyridoxate | 0.045 | 1.233 |  |
| DM | m/z | RT | Name | CpG P | CpG F |  |
| + | 188.103 | 15.4 | guanidinomethyloxopentanoate | <0.001 | 2.182 |  |
| + | 189.087 | 11.2 | N-Acetylglutamine | 0.051 | 0.175 |  |
| + | 189.087 | 14.9 | N-Acetylglutamine | <0.001 | 4.232 |  |
| + | 190.05 | 4.1 | Kynurenate | 0.023 | 1.142 |  |
| + | 190.086 | 10.5 | Indolepropionicacid | 0.013 | 1.349 |  |
| + | 198.087 | 6.3 | N-Acetyl-L-histidine | 0.027 | 0.569 |  |
| + | 198.087 | 8.9 | N-Acetyl-L-histidine | 0.002 | 1.506 |  |
| + | 205.097 | 12 | *Tryptophan | 0.011 | 1.254 |  |
| + | 205.119 | 14.6 | N6-Acetyl-N6-hydroxy-L-lysine | 0.003 | 2.009 |  |
| DM | m/z | RT | Name | CpG P | CpG F |  |
| + | 215.139 | 10.2 | Dethiobiotin | 0.005 | 1.855 |  |
| + | 216.063 | 15.9 | glycerophosphoethanolamine | 0.008 | 1.197 |  |
| + | 217.129 | 15.1 | N-acetyl-(L)-arginine | 0.043 | 0.212 |  |
| + | 220.118 | 8.6 | *Pantothenate | 0.002 | 1.477 |  |
| + | 230.248 | 4.9 | Deoxy-tetradecasphinganine | 0.019 | 0.510 |  |
| + | 240.109 | 12.9 | Dihydrobiopterin | 0.001 | 129.082 |  |
| + | 243.027 | 16.6 | myo-Inositol 1,2-cyclic phosphate | 0.024 | 0.729 |  |
| + | 245.096 | 8.8 | Biotin | 0.045 | 1.453 |  |
| + | 253.144 | 7.5 | ubiquinol-1 | 0.017 | 1.754 |  |
| + | 265.112 | 21.4 | *Thiamine | 0.010 | 1.342 |  |
| + | 276.155 | 17.1 | glutamyl-L-Lysine | <0.001 | 0.551 |  |
| + | 282.279 | 7.5 | Octadecenamide | <0.001 | 0.326 |  |
| + | 298.097 | 7.6 | *5'-Methylthioadenosine | <0.001 | 6.515 |  |
| + | 300.29 | 7.5 | Dehydrosphinganine | <0.001 | 0.331 |  |
| + | 314.269 | 4.2 | N-hexadecanoyl-glycine | 0.008 | 0.489 |  |
| + | 345.185 | 4.3 | Tetracosahexanoic acid | 0.026 | 0.262 |  |
| + | 350.305 | 4.3 | Eicosatrienoyl ethanolamine | 0.049 | 0.072 | |
| + | 354.337 | 4.2 | Eicosaenoyl ethanolamine | 0.041 | 0.152 | |
| + | 377.146 | 8.8 | *Riboflavin | 0.019 | 1.849 | |
| **Fatty acids and phospholipids** | | | | | | |
| - | 253.217 | 3.9 | Hexadecenoic acid | 0.028 | 0.484 | |
| - | 255.233 | 3.9 | Hexadecanoic acid | 0.010 | 0.489 | |
| DM | m/z | RT | Name | CpG P | CpG F | |
| - | 271.228 | 3.9 | 16-hydroxypalmitate | 0.010 | 0.868 | |
| - | 281.249 | 3.9 | Octadecenoic acid | 0.034 | 0.677 | |
| - | 283.264 | 3.9 | Octadecanoic acid | 0.008 | 0.459 | |
| - | 297.243 | 3.9 | Hydroxyoctadecenoic acid | 0.028 | 0.856 | |
| - | 299.259 | 4.0 | Hydroxyoctadecanoic acid | 0.024 | 0.857 | |
| - | 301.217 | 3.9 | Eicosapentaenoic acid | 0.022 | 1.658 | |
| - | 309.28 | 3.9 | Eicosenoic acid | 0.026 | 0.638 | |
| - | 311.223 | 4.0 | Dihydroxyoctadecadienoic acid | 0.039 | 1.343 | |
| - | 311.296 | 3.9 | Eicosanoic acid | 0.009 | 0.433 | |
| - | 319.228 | 4.0 | Hydroxyeicosatetraenoic acid | 0.031 | 1.837 | |
| - | 327.29 | 3.9 | Hydroxyecosanoic acid | 0.002 | 1.428 | |
| - | 333.28 | 3.9 | Docosatrienoic acid | 0.023 | 1.999 | |
| - | 337.311 | 3.9 | Docosenoic acid | 0.015 | 0.469 | |
| - | 339.327 | 3.9 | Docosanoic acid | 0.005 | 0.418 | |
| - | 341.196 | 3.8 | Eicosanedioic acid | 0.009 | 0.872 | |
| + | 452.314 | 4.7 | Lyso PC 14:1 | <0.001 | 0.661 | |
| + | 454.293 | 4.8 | Lyso PE 16:0 | <0.001 | 2.257 | |
| + | 480.308 | 4.7 | Lyso PE18:1 | <0.001 | 2.603 | |
| + | 482.324 | 4.7 | Lyso PE 18:0 | <0.001 | 1.277 | |
| + | 482.361 | 4.9 | Lyso PC 16:1 ether | 0.010 | 0.871 | |
| + | 496.34 | 7.5 | Lyso PC 16:0 | 0.004 | 0.828 | |

| + | 502.291 | 7.5 | Lyso PE 20:4 | <0.001 | 2.128 |
| --- | --- | --- | --- | --- | --- |
| + | 502.293 | 4.7 | Lyso PE 20:4 | <0.001 | 2.249 |
| + | 508.376 | 4.8 | Lyso PC 18:1 ether | <0.001 | 0.576 |
| + | 522.355 | 7.5 | Lyso PC 18:1 | <0.001 | 0.667 |
| + | 524.371 | 4.7 | Lyso PC 18:0 | 0.002 | 0.861 |
| + | 526.293 | 4.6 | LysoPE 22:6 | <0.001 | 3.681 |
| + | 526.313 | 4.3 | LPS 18:0 | <0.001 | 0.406 |
| + | 528.309 | 4.6 | LysoPE 22:5 | <0.001 | 3.304 |
| + | 530.324 | 4.6 | Lyso PE 22:4 | <0.001 | 3.453 |
| + | 538.519 | 4 | SP16:0 | <0.001 | 0.263 |
| + | 544.34 | 4.7 | LysoPC 20:4 | 0.035 | 0.680 |
| + | 552.402 | 4.6 | Lyso PC 20:0 | 0.028 | 1.506 |
| + | 675.544 | 4.5 | SM32:1 | <0.001 | 1.704 |
| + | 689.56 | 4.5 | SM32:0 | <0.001 | 1.358 |
| + | 692.56 | 4.2 | PC30:2 | 0.016 | 0.414 |
| + | 703.575 | 7.5 | SP16:0 | 0.020 | 0.753 |
| + | 703.575 | 4.4 | SP16:0 | <0.001 | 1.365 |
| + | 704.523 | 4.2 | PC32:1 | <0.001 | 20.619 |
| + | 706.539 | 4.2 | PC30:0 | <0.001 | 4.944 |
| + | 716.523 | 4.2 | PE34:2 | 0.001 | 80.073 |
| + | 718.539 | 4.2 | PE34:1 | <0.001 | 8.414 |
| + | 718.575 | 4.2 | PC32:0 ether | 0.007 | 0.896 |
| + | 719.579 | 4.2 | menaquinol-8 | 0.010 | 0.770 |
| + | 720.555 | 4.2 | PE34:0 | <0.001 | 2.140 |
| + | 720.592 | 4.2 | PC32:2 | <0.001 | 0.420 |
| + | 722.513 | 4.1 | PE36:5 ether | 0.003 | 4.012 |
| + | 724.528 | 4.1 | PE36:4 ether | 0.025 | 1.093 |
| + | 728.523 | 4.2 | PC32:3 | <0.001 | 32.232 |
| + | 730.539 | 4.2 | PC32:2 | <0.001 | 11.324 |
| + | 732.554 | 4.2 | PC32:1 | <0.001 | 4.368 |
| + | 732.589 | 4.2 | PE36:0 ether | 0.008 | 0.259 |
| + | 734.497 | 3.9 | PS32:1 | <0.001 | 21.108 |
| + | 734.57 | 4.2 | PC32:0 | 0.002 | 1.414 |
| + | 738.544 | 4.1 | PC34:4 ether | 0.001 | 0.802 |
| + | 740.523 | 4.1 | PE36:4 | <0.001 | 7.660 |
| + | 744.554 | 4.2 | PE36:2 | <0.001 | 15.954 |
| + | 746.57 | 4.2 | PE36:1 | <0.001 | 3.797 |
| + | 746.607 | 4.2 | PC34:0 ether | <0.001 | 1.407 |
| + | 748.528 | 4.1 | PE38:7 | <0.001 | 1.824 |
| + | 749.532 | 4.1 | PG34:1 | <0.001 | 1.886 |
| + | 750.544 | 4.1 | PE38:5 ether | <0.001 | 1.228 |
| + | 751.548 | 4.1 | PG34:0 | <0.001 | 1.220 |
| + | 754.54 | 4.2 | PC34:4 | <0.001 | 16.846 |
| + | 756.555 | 4.2 | PC34:3 | <0.001 | 9.655 |
| + | 758.57 | 4.2 | PC34:2 | <0.001 | 5.430 |
| + | 760.513 | 3.9 | PS34:2 | 0.002 | 7.380 |
| + | 760.586 | 4.2 | PC34:1 | <0.001 | 3.320 |
| + | 762.529 | 3.9 | PS34:1 | <0.001 | 2.360 |
| + | 762.6 | 4.1 | PC34:0 | 0.002 | <0.001 |
| + | 764.524 | 4.1 | PE38:6 | <0.001 | 12.659 |
| DM | m/z | RT | Name | CpG P | CpG F |
| + | 764.544 | 3.8 | PS34:0 | 0.018 | 2.229 |
| + | 766.54 | 4.1 | PE38:5 | <0.001 | 13.705 |
| + | 766.575 | 4.2 | PE36:4 ether | <0.001 | 0.472 |
| + | 768.555 | 4.1 | PE38:4 | <0.001 | 2.268 |
| + | 772.586 | 4.2 | PE38:2 | 0.006 | 8.651 |
| + | 774.544 | 4.1 | PE40:7 ether | <0.001 | 1.903 |
| + | 774.602 | 4.2 | PE38:1 | <0.001 | 2.450 |
| + | 775.547 | 4.1 | PG36:2 | 0.001 | 1.914 |
| + | 776.56 | 4.1 | PE40:7 | 0.001 | 1.499 |
| + | 778.539 | 4.2 | PC36:6 | 0.001 | 29.861 |
| + | 778.576 | 4.1 | PE40:5 ether | 0.006 | 1.169 |
| + | 779.579 | 4.1 | PG36:0 | 0.046 | 1.409 |
| + | 780.555 | 4.2 | PC36:5 | <0.001 | 4.901 |
| + | 780.591 | 4.2 | PE40:4 ether | <0.001 | 0.868 |
| + | 782.57 | 4.2 | PC36:4 | <0.001 | 2.701 |
| + | 784.513 | 3.8 | PS36:4 | <0.001 | 6.632 |
| + | 784.586 | 4.2 | PC36:3 | <0.001 | 4.725 |
| + | 786.529 | 3.9 | PS36:3 | <0.001 | 10.313 |
| + | 786.602 | 4.2 | PC36:2 | <0.001 | 4.253 |
| + | 788.544 | 3.8 | PS36:2 | <0.001 | 1.644 |
| + | 790.56 | 3.9 | PS36:1 | 0.001 | 0.567 |
| + | 792.554 | 4.1 | PE40:6 | <0.001 | 3.914 |
| + | 792.591 | 4.2 | PC38:5 ether | <0.001 | 1.153 |
| + | 794.571 | 4.1 | PE40:6 | <0.001 | 6.982 |
| DM | m/z | RT | Name | CpG P | CpG F |
| + | 796.588 | 4.1 | PE40:4 | 0.004 | 21.543 |
| + | 800.617 | 4.2 | PE40:2 | 0.001 | 4.193 |
| + | 804.554 | 4.2 | PC38:7 | <0.001 | 12.815 |
| + | 806.57 | 4.2 | PC38:6 | <0.001 | 3.835 |
| + | 808.513 | 3.8 | PS38:6 | <0.001 | 16.349 |
| + | 808.586 | 4.2 | PC38:5 | <0.001 | 3.612 |
| + | 809.517 | 3.8 | PI32:2 | <0.001 | 20.365 |
| + | 810.529 | 3.8 | PS38;5 | <0.001 | 6.172 |
| + | 810.602 | 4.2 | PC38:4 | <0.001 | 1.696 |
| + | 811.532 | 3.8 | PI32:0 | 0.001 | 6.252 |
| + | 812.544 | 3.8 | PS38:4 | 0.017 | 1.168 |
| + | 813.685 | 7.4 | SM42:2 | 0.001 | 0.465 |
| + | 813.685 | 4.4 | SM42:2 | 0.009 | 0.475 |
| + | 818.607 | 4.1 | PC40:7 | <0.001 | 1.651 |
| + | 819.518 | 3.7 | PG40:8 | 0.008 | 1.528 |
| + | 820.622 | 4.1 | PC40:5 ether | <0.001 | 1.439 |
| + | 822.638 | 4.2 | PC40:4 ether | <0.001 | 1.216 |
| + | 824.653 | 4.1 | PC40:3 ether | <0.001 | 0.122 |
| + | 828.555 | 4.2 | PC40:7 | 0.003 | 6.93 |
| + | 830.57 | 4.2 | PC40:8 | <0.001 | 2.152 |
| + | 832.586 | 4.1 | PC40:7 | <0.001 | 4.718 |
| + | 834.529 | 3.8 | PS40:7 | <0.001 | 2.931 |
| + | 834.602 | 4.1 | PC40:6 | <0.001 | 2.771 |
| + | 835.532 | 3.8 | PI34:2 | <0.001 | 4.787 |
| DM | m/z | RT | Name | CpG P | CpG F |
| + | 836.545 | 3.8 | PS40:6 | <0.001 | 1.672 |
| + | 836.618 | 4.1 | PC40:5 | <0.001 | 2.173 |
| + | 837.548 | 3.8 | PI34:1 | <0.001 | 1.681 |
| + | 838.56 | 3.8 | PS40:5 | <0.001 | 1.688 |
| + | 838.633 | 4.1 | PC40:4 | <0.001 | 1.616 |
| + | 839.564 | 3.8 | PI34:0 | <0.001 | 1.706 |
| + | 854.57 | 4.1 | PC42:10 | <0.001 | 5.291 |
| + | 856.586 | 4.1 | PC42:9 | <0.001 | 3.356 |
| + | 858.529 | 3.7 | PS42:9 | <0.001 | 3.718 |
| + | 858.602 | 4.1 | PC42:8 | <0.001 | 2.970 |
| + | 859.533 | 3.7 | PI36:4 | <0.001 | 4.644 |
| + | 860.617 | 4.1 | PC42:7 | 0.003 | 10.334 |
| + | 861.548 | 3.7 | PI36:3 | <0.001 | 1.677 |
| + | 862.633 | 4.1 | PC42:6 | <0.001 | 15.191 |
| + | 880.587 | 4.1 | PC44:11 | <0.001 | 18.598 |
| + | 882.602 | 4.1 | PC44:10 | <0.001 | 10.910 |
| + | 884.617 | 4.1 | PC44:9 | <0.001 | 9.615 |
| + | 885.548 | 3.7 | PI 38:5 | <0.001 | 2.817 |
| + | 886.633 | 4.1 | PC 44:8 | 0.001 | 10.089 |
| + | 887.564 | 3.7 | PI 38:4 | <0.001 | 1.795 |
| + | 889.579 | 3.7 | PI 38:3 | <0.001 | 1.433 |

**Table ‎S2**: Metabolites that have significantly changed following BMM exposure to 11a, 12b or 19o prior to CpG treatment versus CpG treatment alone (p<0.05, Students T test, significant differences highlighted in red font). CpG is compared against untreated BMMs. DM refers to detection mode, m/z to mass to charge ratio, F to fold change, RT retention time (min) and P to P-value (n=6). * Indicates retention time corresponding to analytical standard. (PC= phosphatidylcholine, PE= phosphatidylethanolamine, PS = phosphatidylserine, PI= phsophatidylinositol, PG = phosphatidylglycerol, SM = sphingomyelin, SP = sphingosine). ). Data are from a single experiment (n=5 technical replicates) and are representative of two experiments in total.

| DM | m/z | RT | Name | CpG P | CpG F | CpG  11a P | CpG  11a F | CpG  12b P | CpG  12b F | CpG  19o P | CpG  19o F |
| --- | --- | --- | --- | --- | --- | --- | --- | --- | --- | --- | --- |
|  | Choline metabolism | | | | | | | | | | |
| - | 171.006 | 14.8 | * sn-Glycerol 3-phosphate | <0.001 | 1.570 | <0.001 | 0.514 | <0.001 | 0.594 | 0.001 | 1.253 |
| + | 184.073 | 15.3 | *Choline phosphate | <0.001 | 2.338 | <0.001 | 2.413 | <0.001 | 3.075 | 0.001 | 1.299 |
| + | 258.110 | 14.8 | *sn-glycero-3-Phosphocholine | <0.001 | 1.602 | <0.001 | 0.526 | <0.001 | 0.645 | <0.001 | 1.462 |
|  | Creatine metabolism | | | | | | | | | | |
| + | 118.061 | 16.0 | Guanidinoacetate | <0.001 | 2.923 | <0.001 | 0.238 | <0.001 | 0.540 | <0.001 | 2.026 |
| + | 132.077 | 15.0 | *Creatine | <0.001 | 2.499 | <0.001 | 0.613 | <0.001 | 0.625 | <0.001 | 1.734 |
| + | 212.043 | 15.4 | *Phosphocreatine | <0.001 | 3.667 | <0.001 | 0.573 | <0.001 | 0.622 | 0.196 | 1.080 |
|  | TCA Cycle | | | | | | | | | | |
| - | 115.004 | 16.2 | *Fumarate | <0.001 | 2.742 | 0.047 | 1.084 | <0.001 | 1.771 | <0.001 | 1.953 |
| - | 145.014 | 15.7 | *2-Oxoglutarate | <0.001 | 4.484 | <0.001 | 1.211 | <0.001 | 1.895 | <0.001 | 3.144 |
| - | 173.009 | 18.5 | *cis-Aconitate | <0.001 | 2.306 | 0.002 | 1.199 | 0.041 | 2.182 | <0.001 | 7.512 |
| - | 191.020 | 18.4 | *Citrate | <0.001 | 1.776 | 0.002 | 1.261 | <0.001 | 1.544 | <0.001 | 3.237 |
|  | Glutathione metabolism | | | | | | | | | | |
| + | 76.0393 | 16.0 | *Glycine | <0.001 | 1.583 | 0.082 | 0.844 | 0.537 | 0.948 | <0.001 | 4.359 |
| + | 241.031 | 16.6 | L-Cystine | 0.008 | 0.750 | 0.155 | 0.850 | 0.001 | 0.505 | <0.001 | 5.763 |
| + | 251.070 | 14.4 | gamma-L-Glutamyl-L-cysteine | 0.007 | 9.347 | <0.001 | 32.345 | <0.001 | 21.416 | 0.072 | 1.789 |
| + | 308.091 | 14.6 | *Glutathione | <0.001 | 2.170 | <0.001 | 1.818 | <0.001 | 2.190 | <0.001 | 0.245 |
| + | 425.081 | 16.9 | S-glutathionyl-L-cysteine | <0.001 | 3.172 | 0.018 | 1.248 | 0.091 | 1.164 | <0.001 | 4.241 |
| + | 613.160 | 17.6 | *Glutathione disulfide | <0.001 | 4.790 | <0.001 | 1.671 | 0.002 | 1.298 | <0.001 | 0.610 |
| + | 746.099 | 17.5 | NADPH | <0.001 | 3.402 | 0.701 | 0.972 | <0.001 | 1.480 | 0.236 | 1.079 |
|  | Taurine metabolism | | | | | | | | | | |
| + | 110.027 | 15.3 | Hypotaurine | <0.001 | 11.296 | 0.001 | 1.138 | <0.001 | 1.356 | 0.389 | 1.032 |
| + | 126.022 | 15.1 | *Taurine | <0.001 | 3.923 | <0.001 | 1.194 | <0.001 | 1.251 | 0.002 | 1.209 |
| DM | m/z | RT | Name | CpG P | CpG F | C11aP | C11a F | C12bP | C12bF | C19oP | C19oF |
| Carnitine metabolism | | | | | | | | | | | |
| + | 162.113 | 13.6 | L-Carnitine | <0.001 | 1.351 | 0.311 | 0.966 | 0.000 | 0.737 | <0.001 | 2.419 |
| + | 204.123 | 11.3 | O-Acetylcarnitine | <0.001 | 2.784 | 0.918 | 0.996 | 0.004 | 1.172 | <0.001 | 1.571 |
| + | 218.139 | 10.0 | O-Propanoylcarnitine | <0.001 | 1.811 | 0.010 | 0.874 | <0.001 | 0.757 | <0.001 | 0.000 |
| DM | m/z | RT | Name | CpG P | CpG F | CpG  11a P | CpG  11a F | CpG  12b P | CpG  12b F | CpG  19o P | CpG  19o F |
| + | 372.311 | 4.9 | Tetradecanoylcarnitine | <0.001 | 3.079 | 0.036 | 1.077 | <0.001 | 1.670 | 0.189 | 1.092 |
| + | 400.342 | 7.5 | [FA] O-Palmitoyl-R-carnitine | <0.001 | 2.706 | 0.001 | 1.171 | <0.001 | 2.179 | 0.004 | 1.314 |
| + | 426.358 | 4.7 | Elaidiccarnitine | <0.001 | 9.655 | <0.001 | 1.224 | <0.001 | 1.502 | 0.063 | 1.096 |
|  | Sugar Metabolism | | | | | | | | | | |
| - | 111.020 | 8.6 | *Uracil | <0.001 | 2.150 | <0.001 | 0.669 | 0.183 | 0.833 | <0.001 | 9.463 |
| - | 179.056 | 14.7 | *D-Glucose | 0.096 | 0.880 | 0.024 | 0.807 | 0.222 | 0.909 | <0.001 | 6.037 |
| - | 243.062 | 10.0 | *Uridine | <0.001 | 0.691 | 0.008 | 1.208 | 0.220 | 0.921 | <0.001 | 3.526 |
| - | 259.022 | 17.1 | *D-Glucose 6-phosphate | <0.001 | 2.330 | 0.049 | 0.912 | <0.001 | 0.754 | <0.001 | 1.379 |
| - | 289.033 | 16.6 | *Sedoheptulose 7-phosphate | <0.001 | 17.617 | 0.007 | 0.697 | <0.001 | 0.566 | 0.013 | 0.801 |
| - | 323.029 | 15.4 | *UMP | <0.001 | 3.150 | 0.014 | 1.400 | 0.009 | 1.283 | 0.001 | 0.494 |
| - | 324.094 | 14.6 | N-Glycoloyl-neuraminate | <0.001 | 2.672 | 0.034 | 1.078 | <0.001 | 1.272 | <0.001 | 1.514 |
| - | 402.995 | 16.8 | *UDP | <0.001 | 17.538 | 0.008 | 1.247 | <0.001 | 1.569 | 0.226 | 0.893 |
| - | 426.022 | 15.5 | *ADP | <0.001 | 2.816 | 0.003 | 1.123 | <0.001 | 1.443 | 0.168 | 0.927 |
| - | 521.983 | 19.9 | *GTP | 0.002 | 29.142 | 0.028 | 0.458 | 0.760 | 1.070 | 0.003 | 0.121 |
| - | 565.047 | 16.6 | *UDP-glucose | <0.001 | 4.917 | <0.001 | 1.241 | <0.001 | 1.216 | 0.001 | 1.272 |
| - | 579.027 | 19.3 | UDP-glucuronate | <0.001 | 7.065 | 0.001 | 1.147 | <0.001 | 1.309 | 0.003 | 1.304 |
| - | 604.070 | 18.5 | GDP-mannose | <0.001 | 3.358 | <0.001 | 4.389 | <0.001 | 2.134 | 0.040 | 1.638 |
| - | 606.074 | 15.3 | *UDP-N-acetyl-D-glucosamine | <0.001 | 3.680 | 0.002 | 1.116 | 0.004 | 1.099 | 0.013 | 1.160 |
| + | 615.153 | 17.6 | CMP-N-acetylneuraminate | 0.002 | 6.577 | 0.007 | 2.060 | 0.004 | 1.802 | 0.913 | 0.002 |
|  | Lipid Metabolism | | | | | | | | | | |
| + | 216.063 | 15.9 | *sn-glycero-3-Phosphoethanolamine | 0.008 | 1.197 | <0.001 | 0.458 | <0.001 | 0.654 | <0.001 | 1.352 |
| - | 247.058 | 12.8 | Glycerophosphoglycerol | 0.538 | 0.977 | 0.001 | 0.845 | 0.169 | 1.043 | 0.570 | 1.088 |
| + | 300.290 | 7.5 | [SP] 3-dehydrosphinganine | 0.000 | 0.331 | <0.001 | 0.793 | 0.636 | 0.986 | 0.222 | 1.057 |
| - | 311.296 | 3.9 | [FA (20:0)] eicosanoic acid | 0.009 | 0.433 | 0.017 | 0.853 | 0.014 | 0.765 | <0.001 | 0.727 |
| - | 339.327 | 3.9 | Docosanoic acid | 0.005 | 0.418 | 0.021 | 0.798 | 0.130 | 0.862 | 0.096 | 0.798 |
| - | 341.270 | 3.8 | Eicosanedioic acid | 0.009 | 0.872 | 0.001 | 0.912 | 0.004 | 0.946 | 0.010 | 0.944 |
| - | 365.342 | 3.8 | tetracosenoic acid | <0.001 | 0.420 | <0.001 | 0.737 | <0.001 | 0.729 | <0.001 | 1.506 |
| + | 438.298 | 4.7 | Lyso PE 16:1 | 0.274 | 0.939 | <0.001 | 1.252 | <0.001 | 1.385 | <0.001 | 1.664 |
| + | 480.345 | 4.7 | Lyso PC 16:1 | 0.003 | 0.841 | 0.001 | 1.228 | 0.122 | 1.065 | 0.001 | 1.402 |

| DM | m/z | RT | Name | CpG P | CpG F | CpG  11a P | CpG  11a F | CpG  12b P | CpG  12b F | CpG  19o P | CpG  19o F |
| --- | --- | --- | --- | --- | --- | --- | --- | --- | --- | --- | --- |
| + | 104.107 | 20.8 | Choline | 0.000 | 1.567 | 0.012 | 1.121 | 0.003 | 0.801 | 0.000 | 3.573 |
| + | 502.293 | 4.7 | Lyso PE 20:4 | <0.001 | 2.249 | <0.001 | 0.478 | 0.001 | 0.551 | <0.001 | 0.459 |
| + | 526.293 | 4.6 | LysoPE 22:6 | <0.001 | 3.681 | <0.001 | 0.412 | 0.001 | 0.574 | <0.001 | 0.511 |
| + | 528.309 | 4.6 | LysoPE 22:5 | <0.001 | 3.304 | <0.001 | 0.504 | 0.001 | 0.608 | <0.001 | 0.433 |
| + | 530.324 | 4.6 | LysoPE 22:4 | <0.001 | 3.453 | <0.001 | 0.420 | 0.001 | 0.514 | <0.001 | 0.355 |
| + | 706.539 | 4.2 | PC 30:0 | <0.001 | 4.944 | <0.001 | 1.256 | <0.001 | 1.758 | <0.001 | 1.244 |
| + | 718.575 | 4.2 | PC 32:0 ether | 0.007 | 0.896 | <0.001 | 1.214 | <0.001 | 1.329 | 0.001 | 1.136 |
| + | 718.539 | 4.2 | PE 34:1 | <0.001 | 8.414 | 0.001 | 1.172 | <0.001 | 1.498 | 0.051 | 1.069 |
| + | 728.523 | 4.2 | PC 32:3 | <0.001 | 32.232 | <0.001 | 1.276 | <0.001 | 1.358 | 0.133 | 1.081 |
| + | 730.539 | 4.2 | PC 32:2 | <0.001 | 11.324 | <0.001 | 1.241 | <0.001 | 1.491 | 0.022 | 1.106 |
| + | 732.554 | 4.2 | PC 32:1 | <0.001 | 4.368 | <0.001 | 1.145 | <0.001 | 1.389 | 0.014 | 1.076 |
| + | 744.554 | 4.2 | PE 36:2 | <0.001 | 15.954 | 0.001 | 1.102 | <0.001 | 1.412 | 0.772 | 0.993 |
| + | 750.544 | 4.1 | PE 38:5 ether | <0.001 | 1.228 | 0.001 | 1.117 | <0.001 | 1.151 | 0.775 | 0.993 |
| + | 751.548 | 4.1 | PG 34:0 | <0.001 | 1.220 | 0.001 | 1.135 | <0.001 | 1.174 | 0.424 | 1.022 |
| + | 756.555 | 4.2 | PC34.3 | <0.001 | 9.655 | <0.001 | 1.224 | <0.001 | 1.502 | 0.063 | 1.096 |
| + | 758.570 | 4.2 | PC 34:2 | <0.001 | 5.430 | <0.001 | 1.167 | <0.001 | 1.360 | 0.006 | 1.102 |
| + | 766.575 | 4.2 | PC36:4 ether | <0.001 | 0.472 | <0.001 | 1.304 | 0.052 | 1.077 | 0.004 | 1.150 |
| + | 784.586 | 4.2 | PC 36:3 | <0.001 | 4.725 | 0.001 | 1.117 | <0.001 | 1.268 | 0.004 | 1.126 |
| + | 786.602 | 4.2 | PC 36:2 | <0.001 | 4.253 | 0.002 | 1.069 | <0.001 | 1.263 | <0.001 | 1.126 |
| + | 804.554 | 4.2 | PC 38:7 | <0.001 | 12.815 | 0.013 | 1.140 | <0.001 | 1.310 | 0.057 | 1.117 |
| + | 819.518 | 3.7 | PG 40:8 | 0.008 | 1.528 | 0.002 | 0.643 | <0.001 | 0.466 | 0.364 | 0.862 |
| + | 834.529 | 3.8 | PS 40:7 | <0.001 | 2.931 | 0.002 | 0.842 | 0.431 | 0.974 | 0.931 | 1.004 |
| + | 836.545 | 3.8 | PS 40:6 | <0.001 | 1.672 | <0.001 | 0.825 | 0.036 | 0.952 | 0.026 | 0.921 |
| + | 837.548 | 3.8 | PI 34:1 | <0.001 | 1.681 | 0.002 | 0.833 | 0.029 | 0.950 | 0.041 | 0.927 |
| + | 858.602 | 4.1 | PC42:8 | <0.001 | 2.970 | 0.003 | 0.867 | 0.004 | 0.871 | 0.029 | 1.096 |
| + | 861.548 | 3.7 | PI 36:3 | <0.001 | 1.677 | 0.004 | 0.856 | <0.001 | 0.800 | 0.266 | 1.056 |
| + | 863.564 | 3.7 | PI 36:2 | 0.001 | 1.262 | 0.004 | 0.854 | 0.001 | 0.818 | 0.127 | 1.077 |
| + | 865.580 | 3.7 | PI 36:1 | 0.238 | 1.032 | 0.000 | 0.848 | <0.001 | 0.785 | 0.405 | 1.029 |
| + | 882.602 | 4.1 | PC 44:10 | <0.001 | 10.910 | 0.002 | 0.862 | 0.372 | 0.968 | 0.144 | 1.085 |
| + | 884.617 | 4.1 | PC 44:9 | <0.001 | 9.615 | 0.002 | 0.837 | 0.106 | 0.825 | 0.068 | 1.100 |
| + | 885.548 | 3.7 | PI 38:5 | <0.001 | 2.817 | 0.003 | 1.128 | 0.004 | 1.127 | 0.101 | 1.082 |

| DM | m/z | RT | Name | CpG P | CpG F | CpG  11a P | CpG  11a F | CpG  12b P | CpG  12b F | CpG  19o P | CpG  19o F |
| --- | --- | --- | --- | --- | --- | --- | --- | --- | --- | --- | --- |
| Miscellaneous | | | | | | | | | | | |
| + | 134.045 | 15.2 | *L-Aspartate | <0.001 | 1.297 | 0.001 | 1.175 | <0.001 | 1.391 | <0.001 | 2.978 |
| + | 141.066 | 10.5 | Methylimidazoleacetic acid | <0.001 | 2.491 | 0.001 | 0.675 | 0.864 | 1.014 | <0.001 | 2.826 |
| + | 148.060 | 14.9 | *L-Glutamate | <0.001 | 2.248 | <0.001 | 1.180 | <0.001 | 1.412 | <0.001 | 1.897 |
| + | 166.072 | 13.1 | 3-Methylguanine | <0.001 | 178.727 | 0.004 | 1.166 | <0.001 | 1.485 | 0.016 | 1.431 |
| + | 189.087 | 14.9 | *N-Acetylglutamine | <0.001 | 4.232 | 0.001 | 0.838 | <0.001 | 1.307 | <0.001 | 2.246 |
| + | 198.087 | 6.3 | N-Acetyl-L-histidine | <0.001 | 0.569 | 0.002 | 2.299 | 0.038 | 1.708 | 0.001 | 9.038 |
| + | 220.118 | 6.0 | *Pantothenate | <0.001 | 0.484 | 0.003 | 2.810 | 0.127 | 1.903 | <0.001 | 12.844 |
| + | 287.064 | 16.7 | 5'-Phosphoribosylglycinamide | <0.001 | 1377.671 | 0.004 | 0.856 | 0.064 | 0.912 | 0.662 | 1.023 |
| + | 298.097 | 7.6 | *5'-Methylthioadenosine | <0.001 | 6.515 | <0.001 | 1.807 | <0.001 | 3.597 | <0.001 | 3.061 |
| + | 347.040 | 15.7 | *IMP | <0.001 | 3.680 | 0.011 | 1.909 | <0.001 | 8.376 | 0.007 | 0.677 |
| + | 664.117 | 14.4 | *NAD+ | <0.001 | 3.730 | <0.001 | 1.352 | <0.001 | 1.455 | <0.001 | 1.264 |
| + | 666.132 | 13.6 | NADH | 0.000 | 3.715 | 0.600 | 1.053 | 0.001 | 1.525 | 0.010 | 1.395 |

**Table ‎S3**: The list of detected metabolites that have changed following stimulating BMMs with LPS, IFN-γ, IL-4, LPS+IFN-γ and LPS+IL-4. DM refers to detection mode, m/z to mass to charge ratio, RT to retention time (min.) and P to p-value (n=5). * Indicates retention time corresponding to analytical standard. All metabolites detected and listed are from a single experiment. F = fold change in comparison with medium control. ). Data are from a single experiment (n=5 technical replicates) and are representative of two experiments in total.

| DM | m/z | RT | Name | LPS P | LPS F | IFN-γ P | IFN-γ F | IL-4 P | IL-4 F | LPS+IFN-γ P | LPS+IFN-γ F | LPS+IL-4 P | LPS+IL-4 F |
| --- | --- | --- | --- | --- | --- | --- | --- | --- | --- | --- | --- | --- | --- |
| Glycolysis and TCA cycle and related metabolites | | | |  |  |  |  |  |  |  |  |  |  |
| - | 89.024 | 10.0 | *(R)-Lactate | <0.001 | 6.005 | <0.001 | 4.571 | 0.060 | 1.263 | <0.001 | 5.292 | <0.001 | 4.191 |
| - | 117.0193 | 15.6 | *Succinate | 0.001 | 1.452 | 0.003 | 1.292 | 0.803 | 0.965 | 0.150 | 0.861 | 0.003 | 1.574 |
| - | 129.0193 | 15.5 | *Itaconate | <0.001 | 2.128 | <0.001 | 1.606 | 0.599 | 0.887 | <0.001 | 0.387 | <0.001 | 2.435 |
| - | 133.0142 | 16.5 | *(S)-Malate | <0.001 | 1.564 | 0.218 | 1.076 | 0.904 | 0.981 | <0.001 | 2.832 | <0.001 | 1.509 |
| - | 145.0142 | 16.0 | *2-Oxoglutarate | 0.615 | 0.962 | <0.001 | 0.602 | 0.764 | 1.071 | 0.009 | 1.275 | 0.404 | 1.086 |
| - | 168.991 | 15.8 | *dihydroxy acetone phosphate | <0.001 | 8.991 | <0.001 | 6.316 | 0.709 | 1.062 | <0.001 | 11.292 | <0.001 | 5.229 |
| - | 168.991 | 16.6 | *DL-Glyceraldehyde-3-phosphate | <0.001 | 19.947 | <0.001 | 15.927 | 0.001 | 2.425 | <0.001 | 14.700 | <0.001 | 19.725 |
| - | 171.006 | 15.1 | *sn-Glycerol 3-phosphate | <0.001 | 0.667 | 0.001 | 0.717 | <0.001 | 0.234 | 0.063 | 1.175 | <0.001 | 0.493 |
| - | 173.0091 | 18.3 | *cis-Aconitate | <0.001 | 3.164 | <0.001 | 1.975 | 0.648 | 0.900 | 0.507 | 1.087 | <0.001 | 3.484 |
| - | 179.056 | 15.0 | *D-Glucose | <0.001 | 0.452 | <0.001 | 0.395 | 0.022 | 0.709 | <0.001 | 0.336 | <0.001 | 0.501 |
| - | 184.986 | 17.4 | *3-Phospho-D-glycerate | <0.001 | 3.213 | <0.001 | 3.417 | 0.642 | 1.119 | <0.001 | 4.020 | <0.001 | 3.13 |
| - | 213.017 | 15.1 | 2-Deoxy-D-ribose 5-phosphate | 0.004 | 1.668 | <0.001 | 2.487 | 0.473 | 1.170 | <0.001 | 2.583 | <0.001 | 2.323 |
| - | 229.012 | 16.0 | D-Ribose 5-phosphate | <0.001 | 1.709 | <0.001 | 2.524 | 0.337 | 0.867 | <0.001 | 1.871 | 0.077 | 1.154 |
| - | 259.022 | 17.3 | *D-Glucose 6-phosphate | <0.001 | 2.157 | <0.001 | 3.575 | 0.012 | 1.888 | <0.001 | 2.109 | <0.001 | 2.588 |
| - | 289.033 | 16.7 | *Sedoheptulose 7-phosphate | <0.001 | 2.726 | <0.001 | 3.294 | 0.213 | 1.187 | <0.001 | 3.623 | <0.001 | 2.148 |
| - | 338.989 | 18.6 | *D-Fructose 1,6-bisphosphate | <0.001 | 6.323 | <0.001 | 4.607 | 0.150 | 1.369 | <0.001 | 10.520 | <0.001 | 6.034 |
| + | 664.117 | 14.6 | *NAD+ | 0.001 | 1.466 | <0.001 | 1.951 | 0.020 | 1.536 | <0.001 | 1.705 | <0.001 | 1.557 |

| DM | m/z | RT | Name | LPS P | LPS F | IFN-γ P | IFN-γ F | IL-4 P | IL-4 F | LPS+IFN-γ P | LPS+IFN-γ F | LPS+IL-4 P | LPS+IL-4 F |
| --- | --- | --- | --- | --- | --- | --- | --- | --- | --- | --- | --- | --- | --- |
| + | 666.132 | 13.8 | *NADH | <0.001 | 3.004 | <0.001 | 3.042 | 0.958 | 0.992 | <0.001 | 3.251 | <0.001 | 2.937 |
| + | 810.134 | 12.7 | *Acetyl-CoA | <0.001 | 1.832 | <0.001 | 1.782 | 0.002 | 2.166 | <0.001 | 1.586 | <0.001 | 2.227 |
| Oxidative stress | | | |  |  |  |  |  |  |  |  |  |  |
| - | 146.046 | 11.1 | *L-Glutamate | <0.001 | 2.600 | <0.001 | 2.585 | 0.047 | 0.717 | <0.001 | 1.917 | <0.001 | 2.407 |
| - | 166.018 | 9.0 | Homocysteinesulfinicacid | <0.001 | 2.674 | <0.001 | 2.596 | 0.216 | 1.241 | <0.001 | 3.501 | <0.001 | 2.578 |
| - | 249.055 | 14.6 | gamma-L-Glutamyl-L-cysteine | <0.001 | 2.029 | <0.001 | 2.097 | 0.084 | 0.573 | <0.001 | 10.061 | 0.001 | 2.750 |
| - | 465.982 | 17.9 | Deoxy CTP | <0.001 | 3.460 | <0.001 | 4.352 | 0.002 | 3.154 | <0.001 | 7.969 | <0.001 | 5.340 |
| + | 746.099 | 17.5 | *NADPH | 0.714 | 1.051 | 0.002 | 1.511 | 0.040 | 1.517 | 0.504 | 0.920 | 0.008 | 1.407 |
| Taurine metabolism | | |  |  |  |  |  |  |  |  |  |  |  |
| - | 108.012 | 15.7 | Hypotaurine | <0.001 | 2.171 | <0.001 | 2.107 | 0.007 | 1.831 | <0.001 | 2.662 | <0.001 | 2.350 |
| + | 110.027 | 15.6 | Hypotaurine | <0.001 | 1.938 | <0.001 | 1.908 | 0.008 | 1.627 | <0.001 | 2.238 | <0.001 | 2.120 |
| + | 126.022 | 15.6 | *Taurine | 0.015 | 1.237 | 0.004 | 1.263 | 0.955 | 0.993 | 0.002 | 1.373 | 0.004 | 1.317 |
| - | 166.029 | 16.3 | Taurocyamine | 0.097 | 1.431 | 0.001 | 1.501 | 0.049 | 1.413 | <0.001 | 1.680 | <0.001 | 1.762 |
| + | 168.044 | 16.3 | Taurocyamine | <0.001 | 1.568 | <0.001 | 1.527 | 0.148 | 1.264 | <0.001 | 1.640 | <0.001 | 1.719 |
| - | 167.997 | 15.6 | L-Cysteate | 0.028 | 1.271 | 0.005 | 1.295 | 0.876 | 0.977 | 0.002 | 1.457 | 0.005 | 1.340 |
| - | 253.05 | 16.0 | 5-L-Glutamyl-taurine | <0.001 | 4.711 | <0.001 | 8.642 | 0.744 | 0.893 | <0.001 | 4.307 | <0.001 | 4.305 |
| Choline metabolism | | |  |  |  |  |  |  |  |  |  |  |  |
| + | 184.073 | 15.6 | *Choline phosphate | 0.956 | 1.006 | 0.001 | 1.473 | 0.069 | 1.465 | 0.002 | 0.633 | <0.001 | 1.562 |
| + | 258.11 | 15.1 | *sn-glycero-3-Phosphocholine | <0.001 | 0.611 | <0.001 | 0.665 | <0.001 | 0.237 | 0.404 | 1.070 | <0.001 | 0.458 |

| DM | m/z | RT | Name | LPS P | LPS F | IFN-γ P | IFN-γ F | IL-4 P | IL-4 F | LPS+IFN-γ P | LPS+IFN-γ F | LPS+IL-4 P | LPS+IL-4 F |
| --- | --- | --- | --- | --- | --- | --- | --- | --- | --- | --- | --- | --- | --- |
| ATP and high energy phosphates | | | |  |  |  |  |  |  |  |  |  |  |
| + | 212.043 | 15.6 | *Phosphocreatine | 0.001 | 1.413 | <0.001 | 1.425 | 0.002 | 2.082 | 0.002 | 1.371 | <0.001 | 2.294 |
| - | 323.029 | 15.5 | *UMP | 0.857 | 0.957 | 0.062 | 0.650 | 0.199 | 1.252 | <0.001 | 0.273 | 0.155 | 0.743 |
| + | 348.07 | 14.1 | *AMP | 0.010 | 1.364 | 0.002 | 1.443 | 0.033 | 0.683 | 0.204 | 0.862 | 0.318 | 0.907 |
| - | 362.051 | 17.1 | *GMP | <0.001 | 1.708 | 0.000 | 1.930 | 0.071 | 0.770 | 0.012 | 1.321 | 0.037 | 1.208 |
| - | 402.011 | 17.5 | *CDP | 0.043 | 1.397 | 0.001 | 1.535 | 0.567 | 1.113 | <0.001 | 2.427 | 0.004 | 2.085 |
| - | 402.995 | 16.9 | *UDP | 0.325 | 0.876 | 0.018 | 0.740 | 0.062 | 1.409 | 0.010 | 0.695 | 0.081 | 1.354 |
| + | 428.037 | 15.5 | *ADP | <0.001 | 1.592 | 0.004 | 1.261 | 0.209 | 0.864 | 0.001 | 1.458 | 0.005 | 1.298 |
| - | 442.017 | 18.4 | *GDP | <0.001 | 1.522 | 0.011 | 1.235 | 0.202 | 0.773 | 0.024 | 1.294 | 0.019 | 1.376 |
| + | 447.068 | 16.8 | CDP-ethanolamine | 0.006 | 1.311 | <0.001 | 1.751 | 0.077 | 1.323 | <0.001 | 3.662 | <0.001 | 2.010 |
| - | 481.977 | 18.8 | *CTP | 0.757 | 1.033 | 0.002 | 1.455 | 0.169 | 1.255 | <0.001 | 1.968 | 0.001 | 1.665 |
| + | 483.992 | 18.8 | *CTP | 0.920 | 0.989 | 0.004 | 1.376 | 0.178 | 1.256 | <0.001 | 1.809 | 0.006 | 1.462 |
| + | 508.003 | 16.9 | *ATP | 0.063 | 1.181 | 0.032 | 1.217 | 0.335 | 0.874 | 0.064 | 1.200 | 0.769 | 1.030 |
| - | 505.988 | 16.9 | *ATP | 0.065 | 1.175 | 0.020 | 1.226 | 0.271 | 0.856 | 0.088 | 1.165 | 0.863 | 1.018 |
| - | 535.037 | 16.7 | UDP-D-xylose | 0.108 | 1.399 | <0.001 | 1.990 | 0.146 | 1.640 | 0.012 | 1.770 | 0.006 | 2.219 |
| + | 523.998 | 19.7 | *GTP | 0.089 | 1.182 | 0.074 | 1.195 | 0.653 | 0.933 | 0.176 | 1.163 | 0.311 | 1.141 |
| - | 565.047 | 16.8 | *UDP-glucose | 0.008 | 0.737 | 0.002 | 0.720 | 0.164 | 1.261 | 0.206 | 0.891 | 0.842 | 1.017 |
| + | 574.095 | 13.1 | GDP-3,6-dideoxy-D-galactose | <0.001 | 4.762 | <0.001 | 4.918 | 0.625 | 1.118 | <0.001 | 5.678 | 0.002 | 3.490 |
| - | 588.075 | 18.0 | GDP-L-fucose | <0.001 | 1.678 | 0.003 | 1.342 | 0.138 | 0.790 | 0.001 | 1.565 | 0.003 | 1.361 |

| DM | m/z | RT | Name | LPS P | LPS F | IFN-γ P | IFN-γ F | IL-4 P | IL-4 F | LPS+IFN-γ P | LPS+IFN-γ F | LPS+IL-4 P | LPS+IL-4 F |
| --- | --- | --- | --- | --- | --- | --- | --- | --- | --- | --- | --- | --- | --- |
| + | 590.09 | 18.0 | GDP-L-fucose | <0.001 | 1.637 | 0.005 | 1.315 | 0.159 | 0.803 | 0.001 | 1.510 | 0.003 | 1.351 |
| - | 604.07 | 18.7 | GDP-mannose | <0.001 | 2.071 | <0.001 | 2.605 | 0.453 | 0.900 | <0.001 | 3.679 | 0.394 | 1.174 |
| - | 784.149 | 11.8 | FAD | 0.003 | 1.337 | <0.001 | 1.491 | 0.950 | 0.991 | 0.001 | 1.560 | 0.006 | 1.340 |
| Carnitines and carnitine biosynthesis | | | |  |  |  |  |  |  |  |  |  |  |
| + | 162.112 | 13.9 | *L-Carnitine | <0.001 | 0.448 | 0.001 | 0.664 | 0.306 | 1.187 | 0.417 | 0.922 | <0.001 | 0.565 |
| + | 204.123 | 11.6 | *O-Acetylcarnitine | <0.001 | 1.907 | <0.001 | 1.760 | 0.131 | 1.314 | 0.037 | 0.789 | <0.001 | 2.099 |
| Inositol phosphate metabolism | | | |  |  |  |  |  |  |  |  |  |  |
| - | 333.059 | 16.6 | sn-glycero-3-Phospho-1-inositol | <0.001 | 4.694 | <0.001 | 3.092 | 0.002 | 2.140 | <0.001 | 4.057 | <0.001 | 3.261 |
| Propanoate metabolism | | |  |  |  |  |  |  |  |  |  |  |  |
| - | 152.996 | 11.8 | Propanoyl phosphate | <0.001 | 11.827 | <0.001 | 7.023 | 0.696 | 1.147 | <0.001 | 15.509 | 0.001 | 5.510 |
| C5-Branched dibasic acid metabolism | | | |  |  |  |  |  |  |  |  |  |  |
| - | 129.019 | 15.5 | *Itaconate | <0.001 | 2.136 | <0.001 | 1.618 | 0.776 | 1.043 | <0.001 | 0.357 | <0.001 | 2.468 |
| - | 152.996 | 11.8 | Propanoyl phosphate | <0.001 | 11.827 | <0.001 | 7.023 | 0.696 | 1.147 | <0.001 | 15.509 | 0.001 | 5.510 |
| + | 159.076 | 16.6 | 4-Methylene-L-glutamine | <0.001 | 9.083 | <0.001 | 12.743 | 0.600 | 1.036 | <0.001 | 79.755 | <0.001 | 8.048 |
| - | 173.009 | 18.3 | *cis-Aconitate | <0.001 | 3.071 | <0.001 | 1.776 | 0.303 | 1.155 | 0.778 | 0.937 | <0.001 | 3.542 |
| Purine and pyrimidine metabolism | | | |  |  |  |  |  |  |  |  |  |  |
| + | 112.051 | 11.0 | *Cytosine | 0.002 | 0.532 | <0.001 | 0.442 | <0.001 | 0.359 | 0.106 | 0.783 | 0.004 | 0.597 |
| + | 115.05 | 15.3 | 5,6-Dihydrouracil | <0.001 | 1.668 | <0.001 | 1.427 | 0.001 | 2.176 | <0.001 | 1.905 | <0.001 | 2.425 |
| + | 228.098 | 11.0 | *Deoxycytidine | 0.023 | 0.621 | 0.001 | 0.483 | 0.001 | 0.421 | 0.184 | 0.785 | 0.003 | 0.574 |
| + | 129.066 | 15.4 | 5,6-Dihydrothymine | <0.001 | 2.601 | <0.001 | 2.150 | <0.001 | 2.718 | <0.001 | 3.091 | <0.001 | 3.377 |

| DM | m/z | RT | Name | LPS P | LPS F | IFN-γ P | IFN-γ F | IL-4 P | IL-4 F | LPS+IFN-γ P | LPS+IFN-γ F | LPS+IL-4 P | LPS+IL-4 F |
| --- | --- | --- | --- | --- | --- | --- | --- | --- | --- | --- | --- | --- | --- |
| + | 244.093 | 12.5 | *Cytidine | 0.054 | 0.764 | <0.001 | 0.457 | <0.001 | 0.131 | 0.017 | 0.679 | <0.001 | 0.462 |
| - | 243.062 | 10.4 | *Uridine | <0.001 | 0.106 | <0.001 | 0.015 | <0.001 | 0.037 | <0.001 | 0.040 | <0.001 | 0.075 |
| - | 267.073 | 11.4 | *Inosine | <0.001 | 2.469 | 0.001 | 0.361 | <0.001 | 0.051 | <0.001 | 5.189 | 0.197 | 1.240 |
| - | 331.046 | 15.2 | 2'-Deoxyinosine 5'-phosphate | 0.031 | 2.792 | 0.021 | 2.346 | 0.001 | 7.457 | 0.947 | 1.025 | 0.005 | 3.752 |
| - | 285.049 | 17.0 | 5'-Phosphoribosylglycinamide | <0.001 | 185.899 | <0.001 | 104.952 | <0.001 | 314.982 | <0.001 | 64.515 | <0.001 | 334.259 |
| + | 287.064 | 17.0 | 5'-Phosphoribosylglycinamide | <0.001 | 105.701 | <0.001 | 67.499 | <0.001 | 190.202 | <0.001 | 48.502 | <0.001 | 193.545 |
| Aminosugar/glycan metabolism | | | |  |  |  |  |  |  |  |  |  |  |
| - | 178.072 | 11.9 | *D-Glucosamine | 0.094 | 1.125 | 0.002 | 0.817 | 0.001 | 1.659 | 0.005 | 0.765 | <0.001 | 1.545 |
| + | 310.113 | 13.8 | *N-Acetylneuraminate | 0.217 | 1.113 | <0.001 | 0.550 | <0.001 | 0.424 | 0.979 | 1.002 | <0.001 | 0.516 |
| - | 324.094 | 14.9 | N-Glycoloyl-neuraminate | 0.669 | 1.037 | 0.001 | 0.650 | 0.104 | 0.792 | 0.004 | 0.707 | 0.003 | 0.723 |
| + | 590.09 | 18.0 | GDP-L-fucose | <0.001 | 1.637 | 0.005 | 1.315 | 0.159 | 0.803 | 0.001 | 1.510 | 0.003 | 1.351 |
| - | 604.07 | 18.7 | GDP-mannose | <0.001 | 2.071 | <0.001 | 2.605 | 0.453 | 0.900 | <0.001 | 3.679 | 0.394 | 1.174 |
| Arginine metabolism | | |  |  |  |  |  |  |  |  |  |  |  |
| + | 133.0971 | 24.7 | *L-Ornithine | 0.643 | 1.050 | 0.046 | 0.861 | <0.001 | 10.716 | 0.191 | 0.810 | <0.001 | 14.866 |
| + | 146.092 | 15.8 | 4-Guanidinobutanoate | <0.001 | 2.610 | <0.001 | 5.134 | 0.002 | 2.209 | <0.001 | 8.646 | <0.001 | 3.804 |
| + | 176.103 | 16.6 | *L-Citrulline | <0.001 | 10.300 | <0.001 | 13.886 | 0.953 | 1.006 | <0.001 | 84.755 | <0.001 | 9.224 |
| + | 291.13 | 17.3 | N-(L-Arginino)succinate | <0.001 | 5.325 | <0.001 | 4.130 | 0.362 | 0.857 | <0.001 | 30.243 | <0.001 | 6.346 |
| Histidine metabolism | | |  |  |  |  |  |  |  |  |  |  |  |
| + | 141.066 | 9.8 | Methylimidazoleacetic acid | <0.001 | 3.170 | <0.001 | 2.194 | 0.005 | 1.937 | <0.001 | 3.375 | <0.001 | 4.370 |
| + | 141.066 | 10.8 | Methylimidazoleacetic acid | <0.001 | 6.621 | <0.001 | 4.401 | <0.001 | 3.673 | <0.001 | 9.540 | <0.001 | 7.217 |

| DM | m/z | RT | Name | LPS P | LPS F | IFN-γ P | IFN-γ F | IL-4 P | IL-4 F | LPS+IFN-γ P | LPS+IFN-γ F | LPS+IL-4 P | LPS+IL-4 F |
| --- | --- | --- | --- | --- | --- | --- | --- | --- | --- | --- | --- | --- | --- |
| Fatty acids | |  |  |  |  |  |  |  |  |  |  |  |  |
| + | 145.05 | 11.6 | 2,3-Dimethylmaleate | <0.001 | 1.959 | <0.001 | 1.789 | 0.123 | 1.324 | 0.024 | 0.764 | <0.001 | 2.134 |
| - | 158.119 | 11.6 | DL-2-Aminooctanoicacid | <0.001 | 1.898 | <0.001 | 1.803 | 0.101 | 1.350 | 0.017 | 0.77 | <0.001 | 2.122 |
| Creatine metabolism | | |  |  |  |  |  |  |  |  |  |  |  |
| + | 118.061 | 16.5 | Guanidinoacetate | <0.001 | 2.932 | <0.001 | 2.226 | 0.021 | 1.743 | <0.001 | 2.041 | <0.001 | 5.297 |
| + | 132.077 | 15.3 | *Creatine | <0.001 | 1.696 | <0.001 | 1.503 | 0.001 | 2.159 | <0.001 | 2.014 | <0.001 | 2.499 |
| + | 212.043 | 15.6 | *Phosphocreatine | 0.001 | 1.413 | <0.001 | 1.425 | 0.002 | 2.082 | 0.002 | 1.371 | <0.001 | 2.294 |
| Miscellaneous | |  |  |  |  |  |  |  |  |  |  |  |  |
| + | 90.055 | 16.0 | *L-Alanine | <0.001 | 2.519 | <0.001 | 2.493 | 0.001 | 2.316 | <0.001 | 3.467 | <0.001 | 2.86 |
| + | 134.045 | 15.5 | *L-Aspartate | 0.086 | 0.889 | <0.001 | 0.732 | 0.007 | 0.789 | 0.002 | 0.749 | 0.006 | 0.765 |
| + | 156.042 | 15.5 | N-Methylethanolamine phosphate | 0.015 | 1.254 | 0.001 | 1.356 | 0.678 | 0.943 | 0.415 | 0.934 | 0.002 | 1.352 |
| - | 187.072 | 11.0 | *N-Acetylglutamine | 0.003 | 1.570 | 0.004 | 1.434 | 0.59 | 1.085 | <0.001 | 1.992 | <0.001 | 2.768 |
| + | 240.109 | 13.2 | Dihydrobiopterin | <0.001 | 2.223 | <0.001 | 1.723 | 0.255 | 1.219 | <0.001 | 2.277 | <0.001 | 2.476 |
| Phospholipids | |  |  |  |  |  |  |  |  |  |  |  |  |
| - | 154.027 | 15.5 | N-Methylethanolamine phosphate | 0.020 | 1.223 | <0.001 | 1.426 | 0.992 | 1.001 | 0.310 | 1.082 | 0.002 | 1.320 |
| - | 168.043 | 14.6 | Phosphodimethylethanolamine | 0.068 | 1.957 | 0.010 | 3.042 | 0.114 | 2.392 | 0.728 | 1.128 | 0.002 | 2.531 |
| + | 247.058 | 13.1 | Glycerophosphoglycerol | <0.001 | 2.494 | <0.001 | 2.724 | 0.001 | 2.920 | <0.001 | 2.193 | <0.001 | 2.711 |
| + | 258.11 | 15.1 | *sn-glycero-3-Phosphocholine | <0.001 | 0.611 | <0.001 | 0.665 | <0.001 | 0.237 | 0.404 | 1.070 | <0.001 | 0.458 |
